# Evaluation of a GDNF-eluting nanofibrous PCL conduit in a mouse model of peripheral nerve injury

**DOI:** 10.1039/d6ra03291e

**Published:** 2026-05-05

**Authors:** Louis D. V. Johnson, Holly N. Gregory, James B. Phillips, Sara Memarpour Hobbi, Fiona M. Boissonade, Frederik Claeyssens

**Affiliations:** a Kroto Research Institute, School of Chemical, Materials and Biological Engineering, University of Sheffield Broad Lane Sheffield S3 7HQ UK f.claeyssens@sheffield.ac.uk; b School of Clinical Dentistry, University of Sheffield Claremont Crescent Sheffield S10 2TN UK; c The Neuroscience Institute, University of Sheffield Sheffield UK; d Department of Pharmacology, UCL School of Pharmacy London UK; e UCL Centre for Nerve Engineering, University College London WC1E 6BT UK

## Abstract

Severe, gap-type peripheral nerve injuries often require surgical intervention in the form of a nerve autograft or synthetic nerve guidance scaffold to promote axonal regeneration and functional recovery. In this study, nerve guidance conduits (NGCs) were fabricated from aligned polycaprolactone (PCL) nanofibres with or without encapsulated glial cell line-derived neurotrophic factor (GDNF), and a fibrin sealant-based hydrogel. These constructs were evaluated in a murine sciatic nerve transection model using Thy1-YFP-H mice, allowing regenerating axons to be visualised in transverse sections throughout the constructs. Both PCL + GDNF and PCL-only conduits facilitated Schwann cell migration and successful axonal regeneration across the site of injury. Nerve autografts, the positive control, demonstrated the highest regenerating axon count in the distal stump, although statistical significance was not observed between groups. These results demonstrate that NGCs fabricated using aligned PCL nanofibres reliably facilitate nerve regeneration across nerve gaps to a degree, but require further investigation for application in peripheral nerve repair. Future studies that optimise growth factor delivery and conduit design would be beneficial to improve regenerative outcomes.

## Abstract 2

An electrospun nerve guidance conduit comprised of aligned, growth factor-loaded nanofibres and a fibrin hydrogel promotes peripheral nerve regeneration, demonstrating comparable axonal growth to the nerve graft in a severe injury model.

## Graphical abstract

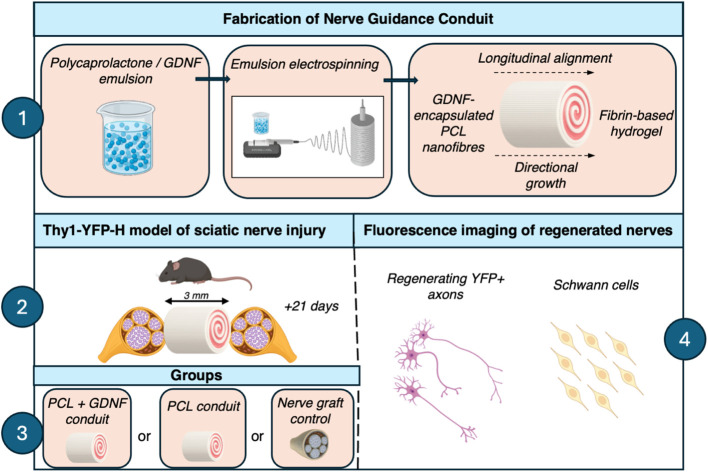

## Introduction

Injuries to the peripheral nervous system (PNS) can result in profound deficits to sensory and motor function, development of neuropathic pain and a consequent reduction in quality of life.^1,2^ Mostly arising from vehicle accidents, peripheral nerve injuries (PNI) can also have iatrogenic causes.^3^ In the case of neurotmesis, the most severe injury, a complete transection of the nerve is observed requiring surgical repair.^4^ Direct end-to-end suturing of the proximal and distal stumps can often result in sufficient regeneration and satisfactory outcomes due to the innate capacity of the PNS to regenerate.^5,6^ However, in gap-type injuries where direct suturing is not possible, nerve autografting remains the gold standard treatment. Autologous nerve grafts supply natural scaffolding and supporting Schwann cells which are crucial for axonal regeneration across the gap.^7^ However, limitations such as donor site morbidity and limited tissue availability mean that alternative therapies are desirable. Nerve guidance conduits (NGCs) enclose the gap between nerve stumps and establish a permissive environment for regeneration. First developed as non-degradable silicone tubes, NGCs have seen substantial technological advancements^8^ and recent clinical trials show efficacy in small digital nerve injuries^9–11^

Nerve regeneration within an NGC, the success of which can vary greatly across species, nerve types and sizes,^11,12^ is highly dependent on the initial formation of a fibrin cable that acts as a bridge between nerve stumps, facilitating infiltration of cells such as Schwann cells prior to axonal growth.^13,14^ Schwann cells, following PNI, undergo a switch from a myelinating to a non-myelinating, pro-regenerative phenotype.^15,16^ In this state, they secrete glial cell line-derived neurotrophic factor (GDNF)^17^ which has a potent effect on axonal outgrowth.^18–21^ However, this regenerative phenotype is transient, and the secretion of GDNF and other neurotrophic factors declines over time,^22,23^ limiting the support available for regenerating axons in the more proximal nerve injuries that must regenerate longer distances before reinnervating their target tissues.

An implantable device that mimics and prolongs the innate response to better facilitate regeneration is highly desirable. Previously, we developed a novel biomaterial consisting of aligned polycaprolactone (PCL) nanofibres with sustained release of GDNF, fabricated *via* emulsion electrospinning.^24^ These nanofibres are similar in diameter to native fibrin fibres,^25^ potentially mimicking the fibrin cable and promoting cellular integration with the material. Furthermore, PCL is an FDA-approved polyester with an appropriate degradation rate for longer nerve gap injuries.^26^ Therefore, this biomaterial is a strong candidate for gap-type peripheral nerve injuries.

NGCs were manufactured by rolling electrospun mats into a cylinder with a spiral cross-section and longitudinal directionality. A hydrogel made from Tisseel (Baxter Healthcare), a commercially available fibrin sealant, was used to as an adhesive to assist with rolling of the nanofibrous mats, as well as maintain conduit patency to allow space for cellular infiltration. A sciatic nerve injury model was utilised using the genetically modified Thy1-YFP-H mouse strain. These mice express yellow fluorescent protein (YFP) within the cytoplasm of a subset of neurons, allowing visualisation of axonal regeneration.^27–29^ This work investigates a novel medical device that combines GDNF-encapsulated PCL nanofibres with a fibrin sealant-based hydrogel, designed to maximise axonal guidance. By rolling the nanofibrous mat, the device maintains a high surface area that provides abundant nanotopographical and biochemical cues for axons to attach and grow, while the fibrin hydrogel preserves the luminal space to prevent obstruction. This combination of structural and biochemical guidance represents a unique approach to address the limitations of current nerve conduits, offering enhanced potential for effective nerve repair.

## Methods

### Electrospinning of GDNF nanofibres

Details of electrospinning conditions for aligned and randomly orientated GDNF-encapsulated nanofibres have been described previously.^24^ Briefly, an emulsion between 12% (w/v) polycaprolactone (PCL) solution in trifluoroethanol and 0.5% (w/v) bovine serum albumin (BSA) solution containing 10 µg recombinant glial cell-line derived neurotrophic factor (GDNF) was prepared using Span-80 (1% v/v) as a surfactant. A Fluidnatek LE-50 instrument (Bioinicia, Spain) was used for electrospinning, and nanofibres were collected on a rotating 100 mm mandrel collector. The 100 mm mandrel collector was rotated at 1750 rpm for 10 minutes, then at 200 rpm for 160 minutes before a further 10 minutes at 1750 rpm. This resulted in a nanofibrous mat that was composed of an aligned/random/aligned tri-layer structure.

### Conduit fabrication

NGCs were fabricated under sterile conditions. First, fibre mats were cut into 3 × 15 mm rectangles that had an average weight of 0.297 mg. Based on our previous work on GDNF release,^24^ an estimated 5.73 ng of protein will be released over the 21 days recovery period. Lyophilised fibrinogen was reconstituted in aprotinin while thrombin (4 UI) was reconstituted in calcium chloride in accordance with the manufacturer's instructions (Tisseel, Baxter), whereafter both components were diluted 1 : 4 in high glucose Dulbecco's modified Eagle medium (DMEM). 50 µL of diluted fibrinogen solution was pipetted onto the fibre mat and left to soak for two minutes. The mat was carefully moved out of the solution to remove excess. Then, 50 µL thrombin solution was pipetted evenly onto the fibre mat and incubated at 37 °C for three minutes. The mat was rolled into a cylinder with a spiral cross-section and further incubated at 37 °C for 15 minutes. Prior to implantation, conduits were subjected to ultraviolet radiation for 30 minutes.

### Scanning electron microscopy

A conduit was snap-frozen in liquid nitrogen for 30 seconds and then freeze-dried for 24 hours. When completely dehydrated, it was transversely sectioned using a scalpel to expose the internal structure. The conduit was mounted onto a stub using carbon tape, gold-coated and imaged using a scanning electron microscope (Inspect F, FEI) where the accelerating voltage and the spot size were 10 kV and 3, respectively.

### Compression testing

Conduits (*n* = 5) were placed horizontally onto a MultiTest-dV tester (Mecmesin, Slinfold, UK). Compression testing of the samples was conducted with a load cell of 25 N which was set at 1 N s^−1^ and a maximum load of 25 N. The compressive Young's modulus was calculated using the linear, elastic region of the stress–strain curves of each sample.

### Implantation into a Thy1-YFP-H mouse sciatic nerve injury model

Thy1-YFP-H mice (YFP+) were obtained from a Home Office approved UK supplier (JAX® mice, Maine, USA *via* Charles River UK Ltd, Margate, UK) and bred in-house. Experiments were carried out under UK Home Office Project and Personal licences, with local ethical approval and in accordance with the Animals (Scientific Procedures) Act 1986. All animal procedures were performed in accordance with the Guidelines for Care and Use of Laboratory Animals of the University of Sheffield and approved by the Animal Ethics Committee of the Animal Welfare and Ethical Review Body (AWERB). The model used for the experiments in this study involved unilateral repair of the sciatic nerve in YFP+ mice. 21 mice between the ages of 6–12 months, male and female, were used (18 YFP+ and 3 C57B/6J (WT)). The experimental groups consisted of a PCL + GDNF conduit (*n* = 6) as described above, a blank PCL control conduit (*n* = 6), and a nerve graft repair (*n* = 6) as positive control, where the graft was obtained from a donor C57B/6J WT mouse. From each donor mouse, 2 donor grafts could be obtained, so 3 WT mice were used for the *n* = 6 autograft group.

For conduit repair groups, YFP+ mice were placed under general anaesthesia (2–3% isoflurane in oxygen; Abbot Laboratories, England) and a single dose of analgesic was administered subcutaneously (Metacam, diluted, 5 mg kg^−1^ dose). The left leg was shaved and cleaned, and the sciatic nerve exposed then transected above the point of bifurcation into tibial and common peroneal nerves. The endings were trimmed to create a gap of 3 mm between the proximal and distal stumps, and either a PCL or PCL + GDNF conduit was sutured end-to-end to the stumps using four 9–0 monofilament polyamide sutures (Ethilon®; Ethicon Ltd, England). Once the nerve endings were secured, muscle and skin were sutured and the mouse was placed in an incubator to recover.

For nerve graft repairs, donor C57B/6J WT mice were anaesthetised using an injectable anaesthetic (ketamine, 100 mg kg^−1^ and medetomidine, 1 mg kg^−1^) and the rear leg shaved and cleaned. The sciatic nerve was harvested and trimmed to 3 mm in length, then sutured into a 3 mm nerve gap in a Thy1-YFP-H mouse as above using four 9–0 monofilament polyamide sutures. Donor mice provided either one or two grafts. Following harvest of grafts donor mice were culled by cervical dislocation whilst under anaesthesia.

### Tissue harvesting and processing

Following a recovery period of 21 days, mice were anaesthetised (ketamine, 100 mg kg^−1^ and medetomidine, 1 mg kg^−1^) and the sciatic nerve/conduit site exposed and freed from surrounding tissue. The skin was sutured to a brass ring to form a pool, which was filled with 4% (w/v) paraformaldehyde for 15 minutes to partially fix the nerve tissues *in situ*. Conduits, along with approximately 2 mm of nerve tissues proximal and distal to the constructs, were removed and fixed in paraformaldehyde for a further 60 minutes. Tissues were then submerged in 30% (w/v) sucrose solution at 4 °C overnight prior to freezing in OCT medium (Agar Scientific, UK), and stored at −80 °C.

A CryoStar NX50 cryostat (Thermo Fisher Scientific) set at −12 °C was then used to collect 14 µm transverse sections from the proximal nerve, conduit entrance, middle of the conduit, conduit exit, and distal stump onto Superfrost Plus™ microscope slides (Thermo Fisher Scientific).

### Haematoxylin and eosin staining

Sections from the middle of either the conduits or nerve grafts underwent haematoxylin and eosin (H&E) staining. The slides were stained using haematoxylin for 1.5 minutes and eosin for 5 minutes, washed with distilled water, dehydrated *via* a series of industrial methylated spririts (IMS) washes and then submerged twice in xylene. Using DPX, the slides were mounted before imaging on an optical microscope using a 4× or 20× objective lens (Motic BA210, China).

### Immunofluorescence

Sections were immunolabelled to show the presence of axons and Schwann cells. Tissue sections were first washed in phosphate-buffered saline (PBS) twice, to remove OCT medium. Sections were permeabilised using 0.1% Triton X-100 in PBS for 15 minutes and then blocked with 3% bovine serum albumin (BSA) solution for 1 hour at room temperature. Sections were washed twice with PBS before the addition of primary antibodies, which were diluted in a 1% BSA solution. Axons were labelled using an anti-β-Tubulin III antibody (ab15708; 1 : 500 dilution) and Schwann cells were labelled using an anti-S100B polyclonal antibody (15146-1-AP; 1 : 500 dilution). Sections were incubated overnight at 4 °C in a humidified chamber. The following day, sections were washed three times with PBS, then incubated with goat anti-rabbit secondary, Alexa Fluor™ 568 (A-11011; 1 : 500 dilution) for two hours at 4 °C. After final PBS washes, slides were mounted using Vectashield® mounting medium with DAPI. Tile-scan images of entire sections were acquired using a fluorescence microscope (THUNDER Imager 3D Cell Culture, Leica Microsystems, Wetzlar, Germany) with a 40× objective. Image acquisition parameters were kept consistent across all samples to enable comparative analysis.

### Image analysis

The total number of YFP+ and β-III tubulin+ (TUBB3+) axons in transverse cross-sections was counted using Fiji software.^30^ Firstly, the images were converted to 8 bit (greyscale), and a threshold with lower and upper limits set at 35 and 255, respectively, was applied. The “Analyse Particles…” tool was used to count objects, providing the total number of axons. The axon count was then expressed as a percentage of the total number of axons in the proximal stump.

For Schwann cell images, fluorescence area was quantified. The images were first converted to 8 bit (greyscale). Because of partial spectral overlap between YFP emission and the red fluorophore channel, the “Image Calculator” tool in FIJI was used to subtract the YFP+ axon signal from the Schwann cell channel to reduce bleedthrough. Fluorescent area and total area were measured, and percentage (%) of fluorescent area was calculated.

### Statistical analysis

Data were analysed using GraphPad Prism 10. Normality of data distribution was assessed using the Shapiro–Wilk test. Two-way analysis of variance (ANOVA) with a multiple comparison Tukey's *post hoc* test was used to compare axon counts between experimental groups and position within the conduit/nerve graft. For Schwann cell fluorescence, a two-way ANOVA (mixed-effects model) was used due to the exclusion of one animal within the PCL conduit group because of technical issues during sectioning and staining. Data were plotted as mean ± standard deviation (SD). Statistical significance was defined where *p* values < 0.05 are considered significant, and denoted as *p* < 0.05 (*), *p* < 0.01 (**) and *p* < 0.001 (***).

## Results

### Fabrication of an implantable conduit from GDNF-encapsulated aligned nanofibres

**Fig. 1:**
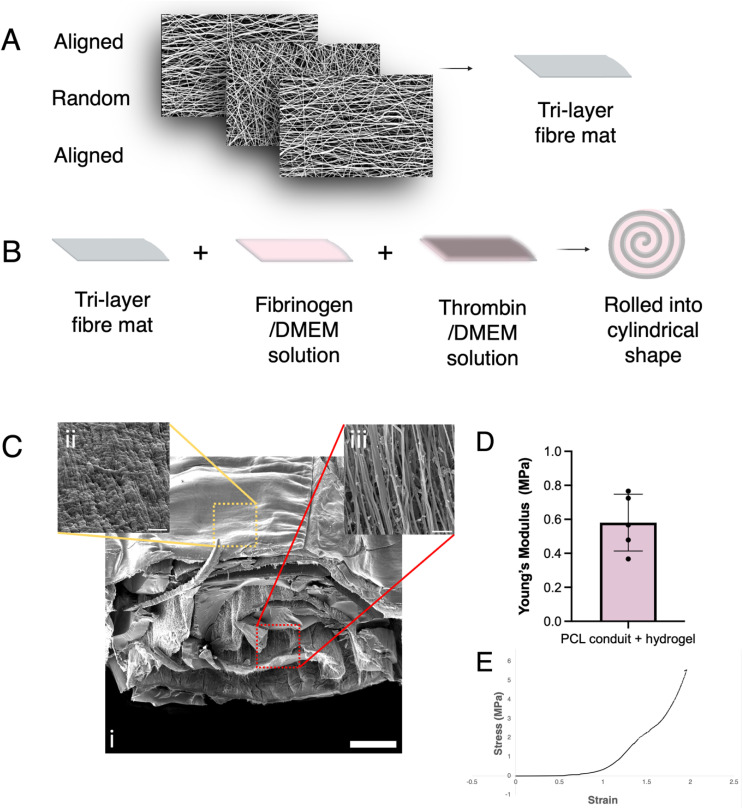
Fabrication and characterisation of nerve guidance conduits. A schematic illustrates how (A) a nanofibrous mat comprised of an aligned-random-aligned tri-layer was (B) rolled into a cylindrical device with a spiral transverse section. Fibrinogen/DMEM and thrombin/DMEM solutions were applied prior to rolling and incubation to allow cross-linking of the hydrogel. (C) Scanning electron micrographs demonstrate the conduit macrostructure after freeze-drying (i) and its outer (ii) and inner (iii) microstructure. After rolling, nanofibres retained their longitudinal alignment and remnants from the lyophilised hydrogel can be observed within the fibres. (D) Compressive Young's modulus of wet conduits (mean ± SD, *n* = 5) and (E) representative stress–strain curve. (Scale bars: C (i) = 4 µm, C (ii) = 2 µm, C (iii) = 200 µm).

Nanofibrous mats with encapsulated GDNF, composed of an aligned-random-aligned tri-layer, were fabricated *via* emulsion electrospinning (Fig. 1A). The mats, approximately 30 µm in thickness, were rolled into cylindrical scaffolds with longitudinal fibre alignment using a diluted fibrin hydrogel (Tisseel) during the rolling process. This hydrogel was intended to both glue the scaffold together and act as a spacer to maintain patency between folds of the mat (Fig. 1B) and the fabricated NGCs had an approximate diameter of 1.2 mm and a length of 3 mm. Following lyophilisation, the resulting conduit was imaged using scanning electron microscopy (SEM), revealing a spiral-like macrostructure in its cross section (Fig. 1C(i)). At higher magnification, the aligned fibrous morphology can be visualised on the outside and inner surfaces of the conduit (Fig. 1C(ii) and (iii)), with remaining proteins of the lyophilised hydrogel observed within the fibrous network. Compressive Young's moduli were calculated using the linear, elastic region of the stress–strain curves of each conduit (Fig. 1D) and mean Young's modulus was 0.58 ± 0.17 MPa.

### Nerve graft/conduit macrostructure and cellular infiltration

**Fig. 2:**
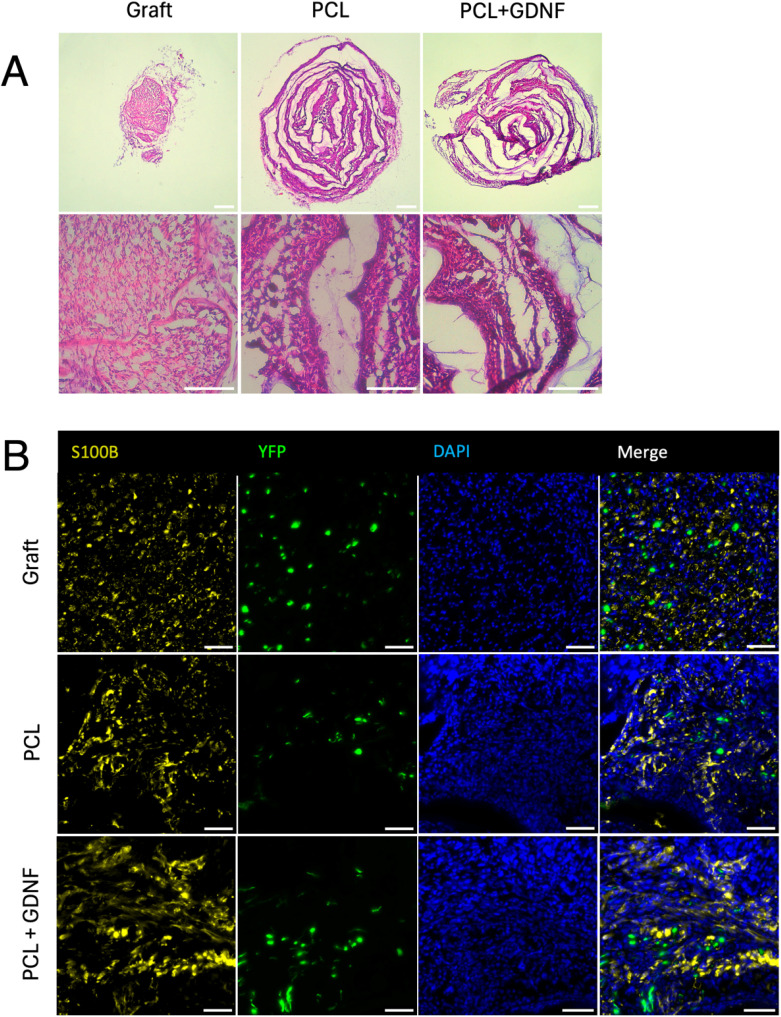
Cellular infiltration into nerve repair constructs. (A) Haematoxylin and eosin (H&E) staining shows macrostructure of nerve graft and conduit transverse sections from middle sections of the scaffolds. Cell nuclei appear dark purple and cytoplasm and extracellular matrix stain pink. (B) Immunofluorescence staining of S100B shows Schwann cells (yellow) occupying space near regenerating YFP+ (green) axons. (Scale bars: A, top = 200 µm, A, bottom = 100 µm, *B* = 50 µm).

Transverse sections of the middle region of constructs (conduits or nerve grafts) were stained using haematoxylin and eosin (H&E) to observe cellular infiltration as well as the macroscopic structure of the nerve graft or conduits (Fig. 2A). Cell nuclei appeared dark purple, cytoplasm and extracellular matrix components were pink and unstained PCL fibres were white, revealing the spiral shape of the rolled fibre mats. The dense cellular presence indicates conduit patency over the 21 days recovery period and cellular infiltration within conduits. Immunostaining showed the presence of S100B+ Schwann cells and regenerating YFP+ axons in both nerve graft and PCL or PCL + GDNF conduits after 21 days recovery (Fig. 2B).

### YFP+ axon regeneration

**Fig. 3:**
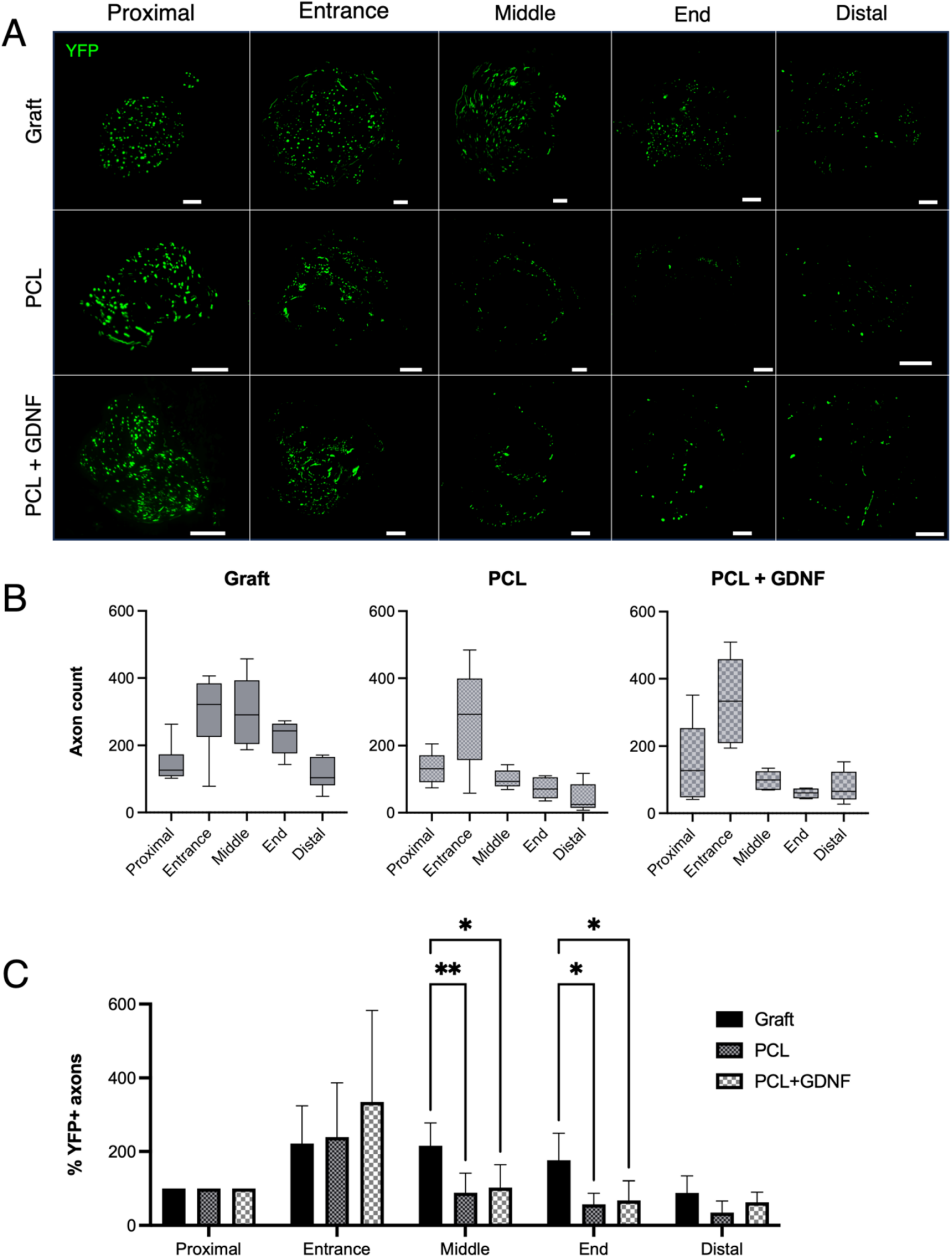
YFP+ axon regeneration through nerve repair constructs. (A) Representative fluorescence images of YFP+ axons regenerating through either nerve grafts or PCL or PCL + GDNF conduits (*n* = 6 per group). (B) Box and whisker plots show median, interquartile range (box), and the minimum and maximum values (whiskers) of total axon counts. (C) Axon counts were normalised to the proximal stump and expressed as a percentage (mean ± SD). Data were analysed with two-way ANOVA with Tukey's *post hoc* test *p* < 0.05 (*), *p* < 0.01 (**) (Scale bars = 200 µm).

Tissue sections of nerve grafts, PCL or PCL + GDNF conduits were collected at the proximal stump, the entrance, middle and end of the constructs, and the distal stump. Fluorescence imaging of these sections allowed visualisation of YFP+ axons, and representative images from each experimental group are shown in Fig. 3A. Box and whisker plots show the median, interquartile range, and the minimum and maximum values of the total YFP+ axon counts within each group (Fig. 3B).

Axon counts within each segment were also expressed as a percentage of the corresponding proximal stump, to account for variability between individual animals (Fig. 3C). A substantial increase in axon number was observed at the entrance to the construct in all groups, indicative of axonal sprouting. In the middle section of the constructs, axon counts dropped markedly in both conduit repair groups, while remaining comparatively high in the nerve graft group. Here, two-way ANOVA with Tukey's *post hoc* multiple comparisons test revealed significant differences between nerve graft and PCL (*p* = 0.009), nerve graft and PCL + GDNF (*p* = 0.025), but not between PCL and PCL + GDNF (*p* = 0.905). Axons counts followed a similar trend at the end of the constructs, with significant differences between nerve graft and PCL (*p* = 0.021), nerve graft and PCL + GDNF (*p* = 0.039), but not between PCL and PCL + GDNF (*p* = 0.910). At the distal stump, the mean axon counts relative to the proximal stump were 88.03 ± 45.79% for the nerve graft, 34.73 ± 31.60% for the PCL conduit, and 62.95 ± 27.16% for the PCL + GDNF conduit, with no statistically significant differences between groups.

### β-III tubulin+ (total) axon regeneration

**Fig. 4:**
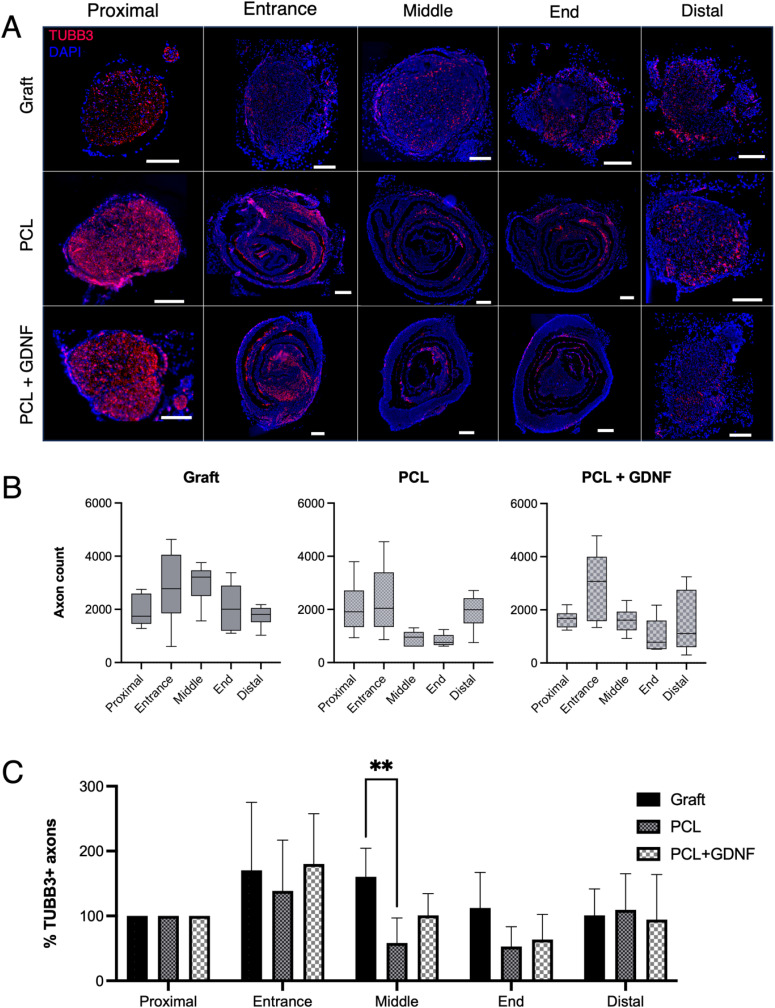
β-III tubulin+ axon regeneration through nerve repair constructs. (A) Representative fluorescence images of TUBB3+ axons regenerating through either nerve grafts or PCL or PCL + GDNF conduits (*n* = 6 per group). (B) Box and whisker plots show median, interquartile range (box), and the minimum and maximum values (whiskers) of total axon counts. (C) Axon counts were normalised to the proximal stump and expressed as a percentage (mean ± SD). Data were analysed with two-way ANOVA with Tukey's *post hoc* test. *p* < 0.01 (**) (Scale bars = 200 µm).

Within the sciatic nerves of Thy1-YFP-H mice, a small percentage of axons are YFP+. Hence, transverse sections were immunolabelled for TUBB3 to quantify the total number of axons at various positions throughout the constructs (Fig. 4A). TUBB3+ axon counts were also presented using box and whisker plots (Fig. 4B) and were normalised as a percentage of the proximal stump (Fig. 4C). The trend observed in YFP+ axon counts was also seen here: axon numbers increased in all groups at the construct entrance, and this higher count was maintained through the middle and end segments of the nerve graft group, but not in the conduit repairs. At the middle segment, Tukey's *post hoc* multiple comparisons test showed that the percentage of axons was significantly higher in the nerve graft than the PCL conduit (*p* = 0.004) but not the PCL + GDNF conduit (*p* = 0.063). Other than this, no other statistical significance was shown. Interestingly, in the conduit repair groups, the number of TUBB3+ axons increased at the distal stump compared to the end segment, a pattern not observed in the YFP+ axon count. Also at the distal stump, the ratio of YFP+ to TUBB3+ is lowest compared to all other segments (Table 1).

**Table 1:** Ratio of YFP+ to TUBB3+ axons within nerve repair constructs

Segment	YFP to TUBB3 axon ratio (%)		
	Graft	PCL	PCL + GDNF
Proximal	7.5	6.4	9.2
Entrance	10.5	12.1	11.5
Middle	10.1	10.8	6.2
End	10.8	8.7	5.8
Distal	6.5	2.3	5.2
**Mean**	9.1 (±2.0)	8.1 (±3.9)	7.6 (±2.7)

### Schwann cells migrated into nerve guidance conduits

**Fig. 5:**
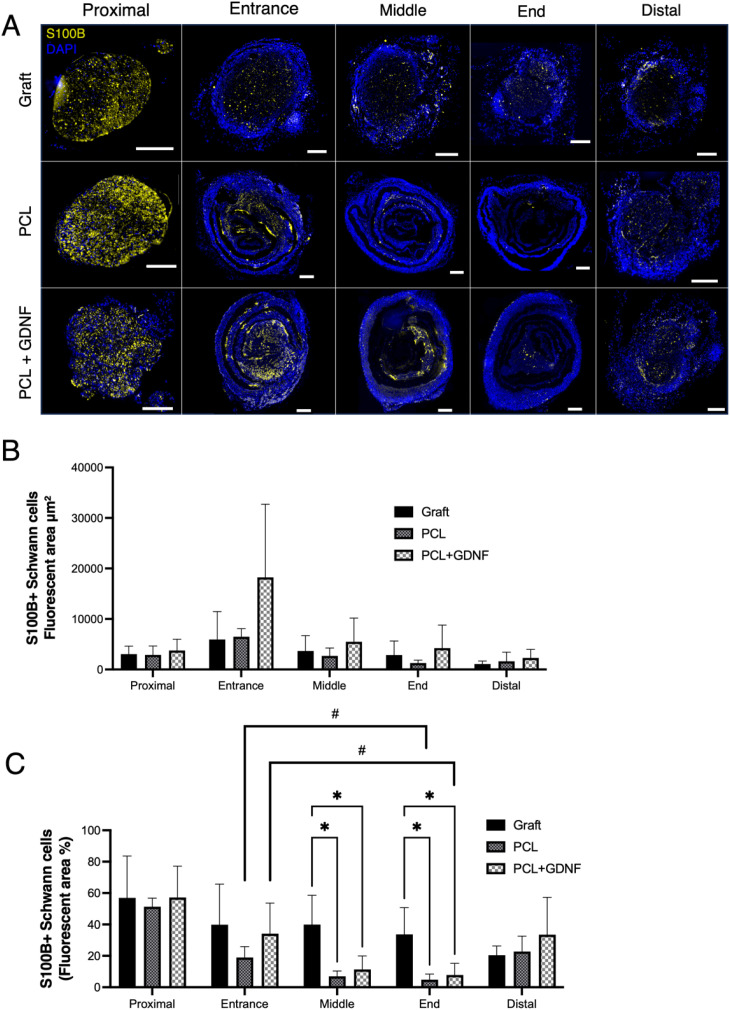
Schwann cell presence within nerve repair constructs. (A) Representative fluorescence images of S100B+ Schwann cells within nerve grafts or PCL or PCL + GDNF conduits. Cells migrated into fibrin hydrogel-filled spaces between nanofibrous mat folds. (B) S100B+ fluorescent area (µm^2^) for Schwann cell quantification. (C) Fluorescent area was normalised to the entire measured area and expressed as a percentage (mean ± SD). Data were analysed by two-way ANOVA with Tukey's multiple comparisons test. Asterisks indicate significant differences between groups within the same segment (*p* < 0.05 (*)), and all such comparisons are shown. Hash symbols (*p* < 0.05 (#)) indicate significant differences between segments within the same group, and all such comparisons are shown, except that comparisons involving proximal segments are omitted. (Scale bars = 200 µm).

In transverse sections throughout each nerve repair construct, Schwann cells were identified using immunostaining for S100B to assess density and position (Fig. 5A). Within the nerve graft sections, the cells presented a homogenous distribution. Within both conduit groups, however, Schwann cell distribution was much more heterogenous, with dense cellular regions observed between the folds of the rolled fibre mat (Fig. 5B). The mean fluorescent area was consistently highest in all positions within the PCL + GDNF group, though two-way ANOVA with Tukey's *post hoc* multiple comparisons test revealed no significant differences between groups. The fluorescent area was also expressed as a percentage of the total cross-sectional area measured (Fig. 5C). The nerve graft group displayed a consistently high Schwann cell density, with two-way ANOVA with Tukey's *post hoc* multiple comparisons test revealing a significantly higher fluorescent area percentage than both conduit groups at middle- and end-sections (*p* > 0.05). In the PCL group, the proximal segment fluorescent area (%) was significantly higher than the entrance (*p* = 0.0065), middle (*p* = 0.0007), end (*p* = 0.0002), and distal (*p* = 0.0305). In the PCL + GDNF group, the proximal segment was higher than the middle (*p* = 0.0260) and end (*p* = 0.0117). No significant differences were observed in the nerve graft group. Interestingly, higher fluorescent area (%) was observed in the conduit entrance compared to the end segments in both the PCL and PCL + GDNF repair groups (*p* = 0.038 and *p* = 0.033, respectively). This indicates that Schwann cells predominantly migrated into the conduits from the proximal rather than the distal nerve stump.

## Discussion

Previously, we demonstrated that alignment of polycaprolactone (PCL) nanofibres directed Schwann cell and axonal orientation *in vitro* while encapsulation of glial cell line-derived neurotrophic factor (GDNF) within the fibres significantly increased axonal outgrowth from primary rat neurons.^24^ This study aimed to assess the effectiveness of a nerve guidance conduit (NGC) fabricated using these fibres *in vivo*.

A key challenge was translating the thin, fibrous mats into NGCs while preserving their high surface area for cellular interactions. Fibrin-based sealants are widely used in surgical coaption of injured peripheral nerves.^31^ Within the context of experimental NGCs, nerve regeneration has been impeded by excessive fibrin sealant-induced cellular infiltration and scar formation.^32^ Additionally, the rapid degradation rate of fibrin-based hydrogels is a concern where mechanical strength is required over a suitable length of time. Yet, using natural polymers in conjunction with synthetic, biodegrable polymers like PCL can prolong scaffold integrity. For example, it was shown that electrospun PCL/collagen nerve conduits mostly degraded over a 4 months period in rats^33^ while another showed minimal foreign body response and minimal of subcutaneously-implanted pure PCL fibre meshes.^34^ Furthermore, a fibrin sealant, diluted in cell culture medium, has been previously optimised to support sensory axonal growth.^35^ In our study, this hydrogel served as both an adhesive, enabling the mats to be rolled into a cylinder with a spiral cross-section, and a spacer which allowed patency of the conduit with maximum surface for regenerating axons and cellular infiltration. We propose the NGCs within this study would likely degrade slowly enough to allow more complete regeneration over the nerve gap before degrading safely in longer follow-up studies. Yet, the authors acknowledge the limitation of using a human-derived fibrin glue in a murine model, as differences in fibrinolytic activity vary across species and potential xenogeneic immune responses may accelerate scaffold degradation and influence the regenerative microenvironment.

The NGCs comprised a nanofibrous mat comprised of a tri-layer with aligned/random/aligned orientation, which exploits distinct advantages of both types of material. Sheets comprising of random fibres have superior tensile strength than their aligned counterparts as demonstrated previously in PCL^36^ and PCL/chitosan^37^ fibres, which is likely advantageous in avoiding tearing issues during suturing. In addition, randomly oriented fibres resulted in a 20% more efficient encapsulation of bovine serum albumin compared to aligned fibres,^38^ while an ibuprofen-loaded PCL tri-layer scaffold has demonstrated a more sustained release profile.^39^ Therefore, a random fibre layer was an attractive option. However, the outer aligned component of the tri-layer was necessary to facilitate better directed axonal/cellular growth which we have shown previously.^24^ Scanning electron micrographs of the conduits demonstrated that the nanofibres retained their longitudinal alignment after manufacture. While NGCs that are far stiffer than the native environment can cause compressive damage to the surrounding tissue and excessive inflammation,^40,41^ those that are too soft risk collapse and kinking, detrimental to nerve regeneration.^42^ It is significant that these NGCs reliably facilitated cellular infiltration and axonal growth throughout the scaffolds while demonstrating a lower compressive modulus than that of NGCs within previous studies, which have ranged from 3.2 MPa^28^ to 470 MPa.^43^

This study utilised a 3 mm mouse sciatic nerve gap as a model of peripheral nerve injury. Although an injury of this size is relatively small compared to those used in other murine studies,^44,45^ smaller gap models remain useful in capturing the initial stages of axonal growth.^46^ Furthermore, a small gap of 4 mm in a murine model bridged by a non-porous or micro-porous gelatin methacryloyl (GelMA)-based NGC resulted in similar functional recovery after a 4 months recovery period,^47^ highlighting the usefulness for earlier comparison within these models. Moreover, the study utilised the Thy1-YFP-H strain of mice, which express YFP in a subset of motor and sensory neurons allowing visualisation of regenerating axons.^27^ The number of regenerating axons was counted throughout the constructs and in the distal nerve stump, a method reliably used to quantify nerve regeneration.^48–51^ Our results showed the highest mean axon count within the distal stump was within the nerve graft group, which was as expected given the well-established efficacy of nerve grafts as a clinical gold standard. The PCL + GDNF conduit showed a promising regenerative response, although statistical analysis indicated no differences between this group and PCL-only. This finding could be attributed to suboptimal GDNF dosage: within this study, the concentration of GDNF within PCL nanofibres was based on previous results which saw significant improvements in neurite length *in vitro*.^24^ However, this may not reflect the optimal concentration *in vivo*, and future studies could indeed investigate this optimal dosage for improved results. However, this must be carefully considered as prolonged high concentrations of this therapeutic agent may cause a ‘candy store’ effect in which axons preferentially remain in the conduit rather than progress into the distal stump.^52–54^ Another study has previously utilised biodegradable microcarriers to ensure a sustained delivery of neurotrophic factors including GDNF,^55^ protecting the protein from intrinsic proteases which contribute to a fast degradation rate *in vivo*. Indeed, short half-lives and the potential for irreversible binding to biomaterial surfaces can decrease the effectiveness of growth factors which can further obscure any investigation into an optimal dosage.

Within the nerve graft group, a gradual decrease in YFP+ axon numbers was observed throughout the constructs and into the distal stump. This differed from the pattern observed in both conduit groups, where an initial high axon count at the entrance (likely due to axonal sprouting following transection of the proximal stump^56,57^) was followed by a sharp decrease at the middle segment of the constructs. Moreover, at the middle and end segments, the number of axons was significantly higher in the nerve graft than both conduit groups. This is likely due to an abundance of organised Schwann cells, pre-existing vasculature and intact endoneurial tubes that provide an uninterrupted pathway throughout for regenerating axons that together facilitate the optimal environment for axonal regeneration within nerve grafts.^58,59^ Though missing some of these factors, Schwann cells were also abundant within the conduits, with the highest fluorescence area (µm^2^) observed within the PCL + GDNF conduits at all segments (although no statistical significance was observed between groups). However, as conduit diameters were much larger than those of the nerve grafts, normalisation revealed that Schwann cells were significantly more dense within the nerve graft group at the middle and end segments compared to conduit groups.

While the use of Thy1-YFP-H mice allowed visualisation and robust quantification of axonal regeneration within a subset of axons, transverse sections were additionally stained for β-III tubulin (TUBB3) to allow visualisation of all neurites within the constructs. The amount of YFP+ axons in this strain was originally qualitatively estimated as ‘few’ in motor neurons and ‘many’ in the dorsal root ganglia (DRG).^27^ Another study estimated the ratio of YFP to total sensory axons to be 2.6% in the common fibular nerve, based on assumptions on the previously reported axon counts within the DRG.^60^ In the present study, the ratios of YFP+ to TUBB3+ axons within the regenerating sciatic nerve was provided. This information is valuable, validating the use of YFP fluorescence as a proxy for estimates of overall axonal regeneration. Nonetheless, the TUBB3+ axon count showed a similar trend to the YFP+ count across all but one position: the distal stump, where axon counts markedly increased in both conduit groups. Wallerian degeneration, the process in which in which axonal fragments and myelin debris are phagocytosed, is well documented.^61–63^ However, the exact rate at which this occurs is not clear. One explanation could be the presence of remnant TUBB3 fragments within the distal stumps that have not yet been phagocytosed or degraded, that are able to be recognised by anti-TUBB3 antibodies, leading to fluorescent staining even in the absence of intact, regenerating axons.

Schwann cell presence was higher at conduit entrance rather than end segments in both conduit groups, indicating they predominantly migrated from the proximal stump. This is consistent with another study that observed far fewer Schwann cells migrating from the distal compared to the proximal stump into a 5 mm-long gap following sciatic nerve transection in mice.^64^ Schwann cells are crucial for successful nerve regeneration due to their ability to dedifferentiate, proliferate, and elongate into bands of Büngner which provide structural and molecular cues for the regenerating axons^15,16,22^ and so their migration into the conduits may have been critical to successful axonal growth throughout the constructs.

## Conclusion

This study demonstrates that PCL nanofibre-based nerve guidance conduits support axonal regeneration and Schwann cell infiltration *in vivo*. The use of Thy1-YFP-H mice enabled direct visualisation of regenerating axons, with supplementary β-III tubulin staining confirming that YFP fluorescence is a reliable proxy for overall axonal regeneration. Schwann cell migration into the constructs highlights the importance of cellular guidance in successful nerve regeneration. Future work should focus on optimising GDNF dosage to further improve regenerative outcomes. Nonetheless, this study supports the potential of aligned, GDNF-loaded nanofibre conduits as a useful biomaterial component for consideration in the development of alternatives to autografts for peripheral nerve repair.

## Author contributions

LJ: conceptualization, formal analysis, investigation, methodology, writing – original draft, writing. HG: conceptualization, funding acquisition, formal analysis, investigation, methodology, writing – review and editing. SMH: supervision. JP: conceptualization, funding acquisition, supervision, writing – review and editing. FB: conceptualization, funding acquisition, supervision, writing – review and editing. FC: conceptualization, funding acquisition, supervision, writing – review and editing.

## Conflicts of interest

There are no conflicts to declare.

## Supplementary Material

RA-016-D6RA03291E-s001

RA-016-D6RA03291E-s002

RA-016-D6RA03291E-s003

RA-016-D6RA03291E-s004

## Data Availability

The data supporting this article have been included as part of the supplementary information (SI). Supplementary information is available. See DOI: https://doi.org/10.1039/d6ra03291e.
