# Supplementary material for: Evaluation of a GDNF-eluting nanofibrous PCL conduit in a mouse model of peripheral nerve injury
Source: RSC Adv. 2026 May 5;16(25):23177–89. doi: 10.1039/d6ra03291e (PMC13141687; doi:10.1039/d6ra03291e)
Supplement: RA-016-D6RA03291E-s002 [file RA-016-D6RA03291E-s002.pdf]

Supplementary Information (SI) for RSC Advances.
This journal is © The Royal Society of Chemistry 2026

Supplementary material

Supplementary table 1. Mean YFP+ and TUBB3+ axon counts from different positions within nerve repair constructs.

	Mean axon count (± SD)					
**Segment**	**Graft YFP**	**Graft TUBB3**	**PCL YFP**	**PCL TUBB3**	**PCL+GDNF YFP**	**PCL+GDNF TUBB3**
Proximal	145 ± 60	1929 ± 587	133 ± 47	2065 ± 995	153 ± 119	1655 ± 332
Entrance	296 ± 117	2819 ± 1407	282 ± 147	2333 ± 1296	337 ± 127	2943 ± 1292
Middle	301 ± 101	2987 ± 761	100 ± 27	923 ± 282	99 ± 28	1605 ± 479
End	225 ± 51	2076 ± 877	73 ± 34	832 ± 242	60 ± 15	1032 ± 651
Distal	114 ± 47	1754 ± 397	44 ± 43	1913 ± 668	78 ± 48	1506 ± 1177

Images of YFP+ and β-III tubulin+ axons of a control (uninjured) nerve were taken prior to undertaking the study. The baseline fluorescence for both YFP and β-III tubulin expression are similar to what was observed in the proximal stump of the injured sciatic nerve.

BF: 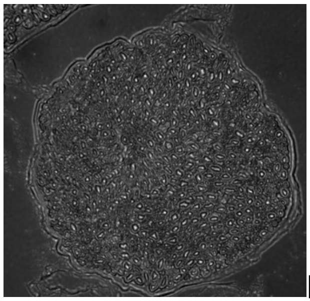 DAPI: 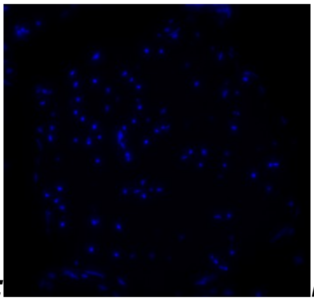 ***B3T:*** 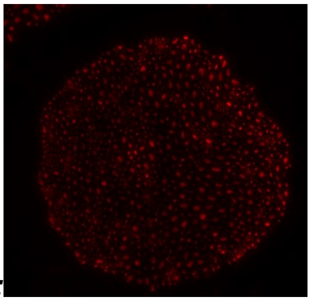

YFP: 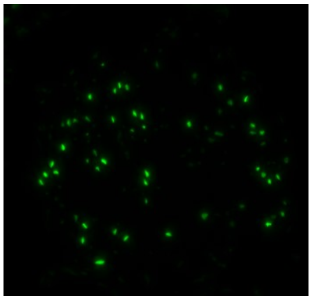

Supplementary table 2. Defining characteristic of experimental mice.

MOUSE TYPE	D.O.B	INTERVENTION	DATE OF SURGERY	AGE AT TIME OF SURGERY (MONTHS)	SEX
**THY1-YFP-H**	22/01/2024	PCL	21/01/2025	11	F
**THY1-YFP-H**	12/02/2024	PCL	21/01/2025	11	F
**THY1-YFP-H**	10/03/2024	PCL	21/01/2025	10	F
**THY1-YFP-H**	12/05/2024	PCL	12/03/2025	10	M
**THY1-YFP-H**	12/05/2024	PCL	12/03/2025	10	M
**THY1-YFP-H**	12/09/2024	PCL	12/03/2025	6	M
**THY1-YFP-H**	18/04/2024	PCL/GDNF	17/03/2025	10	M
**THY1-YFP-H**	18/04/2024	PCL/GDNF	17/03/2025	10	M
**THY1-YFP-H**	18/04/2024	PCL/GDNF	17/03/2025	10	F
**THY1-YFP-H**	18/04/2024	PCL/GDNF	17/03/2025	10	F
**THY1-YFP-H**	18/04/2024	PCL/GDNF	17/03/2025	10	F
**THY1-YFP-H**	10/03/2024	PCL/GDNF	12/03/2025	12	F
**THY1-YFP-H**	10/03/2024	Graft	11/03/2025	12	F
**THY1-YFP-H**	18/04/2024	Graft	11/03/2025	10	F
**THY1-YFP-H**	03/06/2024	Graft	10/03/2025	9	F
**THY1-YFP-H**	13/05/2024	Graft	10/03/2025	9	F
**THY1-YFP-H**	18/04/2024	Graft	11/03/2025	10	M
**THY1-YFP-H**	18/04/2024	Graft	11/03/2025	10	M
**C57B/6J**	24/02/2024	Donor	10/03/2025	12	F
**C57B/6J**	24/02/2024	Donor	11/03/2025	12	F
**C57B/6J**	24/02/2024	Donor	11/03/2025	12	F

	PCL	PCL/GDNF	Graft
**AGE (MEAN)**	9.66666667	10.33333333	10
**SD**	1.86189867	0.816496581	1.095445115
